# Spontaneous Rupture of a Hepatic Artery Aneurysm: A Case Report, Against the Odds

**DOI:** 10.5811/cpcem.42509

**Published:** 2025-10-22

**Authors:** Vahe Zograbyan, Andrea Hladik, Lysdie Espinoza, Manuel Cruz

**Affiliations:** Eisenhower Health, Department of Emergency Medicine, Rancho Mirage, California

**Keywords:** hepatic artery aneurysm, coil embolization, hepatobiliary, abdominal pain, case report

## Abstract

**Introduction:**

Ruptured aneurysms are associated with significant mortality limiting a patient’s chances of survival, making early and accurate diagnoses crucial. A commonly overlooked cause is the hepatic artery aneurysm, where most patients exhibit no distinct symptoms and detection typically occurs only after the aneurysm has ruptured. Hepatic artery aneurysms are linked with high rupturing rates resulting in substantial mortality when compared to other splanchnic artery aneurysms. Enhancing recognition and consideration of splanchnic artery aneurysms, including hepatic artery aneurysms, will increase a patient’s odds of a successful recovery. The following case report illustrates the critical nature of these cases and highlights how important early diagnosis and aggressive intervention are to prevent death once rupture of the hepatic artery aneurysm has occurred.

**Case Report:**

A 57-year-old female presented to the emergency department brought in by helicopter for generalized chest and abdominal pain. A computed tomography angiography of the chest, abdomen, and pelvis was performed and revealed a saccular aneurysm exhibiting multiple lobes in the left hepatic artery accompanied by hemoperitoneum confirming a spontaneous rupture. As a result of the ruptured aneurysm, it was decided an immediate coil embolization was necessary. Ultimately the patient underwent a successful coil embolization and was transferred to a facility with hepatobiliary and transplant surgery capabilities. She remained stable, was extubated the following day, and did not require any additional surgeries.

**Conclusion:**

By encompassing hepatic and associated splanchnic artery aneurysms in the diagnosis of patients with abdominal pain and signs of hemodynamic instability, physicians can improve early identification, facilitating early endovascular repair and improved patient outcomes. It is a rare diagnosis that can present with a wide range of symptoms. Currently, endovascular approaches for ruptured hepatic artery aneurysms are preferred over open surgery.

## INTRODUCTION

Most intra-abdominal aneurysmal ruptures have catastrophic consequences; as a result, emergency physicians often have a heightened suspicion for rupture in patients who present hypotensive with abdominal pain. A rapid but detailed history and thorough physical exam are necessary in pointing the practitioner in the direction of expedient diagnosis and subsequent definitive treatment. While repair is clearly mandated in patients with a symptomatic aneurysm or contained rupture, asymptomatic lesions also warrant intervention. Splanchnic artery aneurysms often have no specific symptoms and are found incidentally, making the decisions regarding treatment difficult.^1^ The natural clinical course of these aneurysms has not been well defined.^2^ Although hepatic artery aneurysms are rare, they have an overall reported rupture rate of 44%.^3^ Because of the high mortality associated with emergent repair, aggressive treatment is necessary in symptomatic patients. The following case report focuses on a case in which rapid detection and aggressive intervention saved the patient’s life.

## CASE REPORT

A 57-year-old female with unknown past medical history presented to the emergency department (ED) via helicopter air ambulance with complaints of chest and epigastric pain since the previous night. Per emergency medical services on scene, the patient was found to be tachycardic, diaphoretic, ill-appearing, and somnolent; given the extended ground transport times, air ambulance transport was called. En route, given the complaint of chest pain since the previous night, there were concerns for possible cardiac etiology of the patient’s condition and she was given aspirin, nitroglycerin, and fentanyl, although there were no acute ischemic changes noted on her electrocardiogram (ECG). On arrival to the ED, the patient was pale, cool, diaphoretic, tachycardic with a heart rate of 123 beats per minute, hypotensive with a blood pressure reading of 92/75 millimeters of mercury, and oriented only to person and time. Her exam findings included mid-range and reactive pupils, clear lungs, normal heart sounds, and a soft abdomen.

**Image 1:**
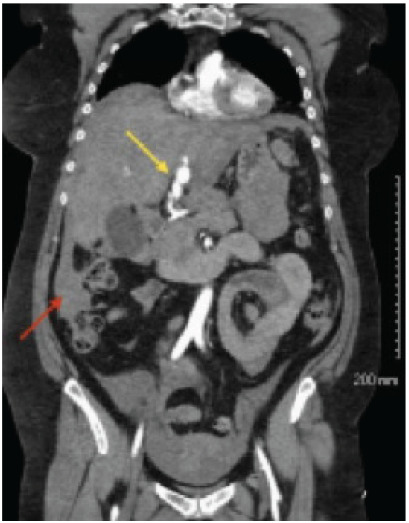
Computed tomography angiography of the chest, abdomen, and pelvis of a patient with abdominal pain and hypotension, showing a bilobed, multilobulated saccular left hepatic artery aneurysm (yellow arrow) and hemoperitoneum (red arrow).

Bilateral, large-bore peripheral intravenous lines were established, and laboratory studies including type and screen, basic chemistries, complete blood cell count, high sensitivity troponins, lactic acid, blood cultures, arterial blood gas, pregnancy test, lipase, and urinalysis were collected. Crystalloid fluid boluses were initiated. Point-of-care blood glucose demonstrated euglycemia, and a 12-lead ECG was unremarkable for any acute ischemic changes. Bedside extended focused assessment with sonography for trauma demonstrated good lung sliding bilaterally, no cardiac tamponade, and no appreciable free fluid in the abdomen. The patient then underwent a non-contrast computed tomography (CT) of the head and CT angiography of the chest, abdomen, and pelvis to help identify the etiology of her symptoms. Differential diagnoses included aortic dissection, ruptured abdominal aortic aneurysm, massive pulmonary embolism, and septic shock. The patient was found to have a bilobed multilobulated saccular aneurysm arising from the left hepatic artery measuring 1.7 x 1.6 x 2.1 cm and 1.1 x 1.0 x 1.9 cm with hemoperitoneum consistent with rupture (Image 1).

**Image 2:**
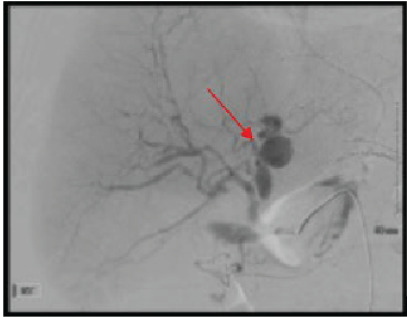
Angiography of the multilobulated hepatic artery aneurysm (red arrow) seen under fluoroscopy in a 57-year-old female with abdominal pain and hypotension.

The patient’s initial hemoglobin was measured at 11 grams/deciliter (g/dL (reference range: 12–16 g/dL); however, given hemoperitoneum and signs of shock, a single lumen 9 French central line was established, and the patient was started on massive transfusion protocol. After consultation with interventional radiology, vascular surgery, and general surgery, the patient was taken emergently to the interventional radiology (IR) suite for angiography (Image 2) and coil embolization.

*CPC-EM Capsule*

What do we already know about this clinical entity?

*Splanchnic artery aneurysms tend to be asymptomatic until time of rupture and have a high mortality rate*.

What makes this presentation of disease reportable?

*We report the case of a patient with early signs of shock and ruptured hepatic aneurysm, whose diagnosis was made rapidly and led to emergent intervention*.

What is the major learning point?

*Rapid diagnosis and early resuscitation is critical. Although the patient ultimately did well, more aggressive treatment could have been initiated in this case*.

How might this improve emergency medicine practice?

*Given the high mortality rate, aggressive and expedient resuscitation, such as transfusion, should be considered in the treatment of splanchnic artery aneurysms*.

**Image 3:**
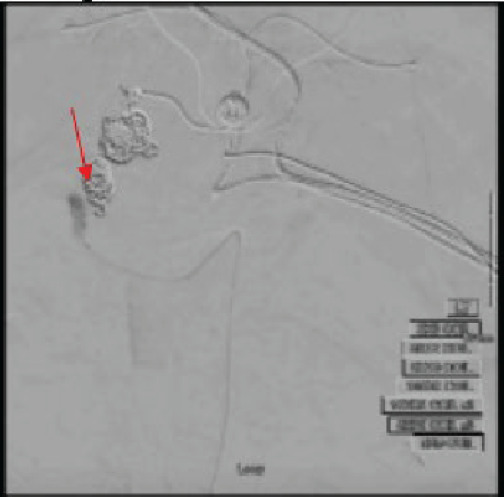
Embolization of the multilobulated hepatic artery aneurysm with 11 coils (red arrow) placed by interventional radiology, as seen under fluoroscopy.

Coiling was completed with 11 coils (Image 3) to the left hepatic artery aneurysm, which appeared to control the bleeding.

However, during the IR procedure, the patient became profoundly hypotensive, necessitating treatment by the ED team with a push dose of 100 micrograms (mcg) of epinephrine to stabilize her blood pressure prior to transfusion while transporting her back to the ED. During transport her blood pressure continued to be in the range of 60/42 mm Hg with a heart rate of 147 bpm, and it was decided to give a second emergent push dose of epinephrine (100 mcg) until the blood transfusion could be started. She developed a worsening of her mental status with stupor and was eventually intubated for airway protection upon return to the ED. Given the location of the aneurysm and the lack of availability of hepatobiliary or transplant surgery specialties at our facility, the patient, once stabilized, was ultimately transferred to a facility with such capabilities. If she had developed further bleeding, liver mobilization with transection of the hepatic artery would had to have been considered. However, after transfer the patient remained stable, with no further bleeding, and was extubated the following day. She did not require any further surgeries as the coils successfully embolized the ruptured hepatic artery aneurysm.

## DISCUSSION

Splanchnic artery aneurysms are more often found in autopsy studies as compared with abdominal aortic aneurysms, yet aortic aneurysms are repaired much more frequently. Up to 10% of autopsy reports find splanchnic artery aneurysms, whereas only 0.5% find abdominal aortic aneurysms.^4^ Early recognition of splanchnic artery aneurysms is imperative as nearly one in four may be complicated by rupture with mortality approaching 80–100% after rupture.^5^ Among splanchnic arteries, aneurysms of the splenic artery are the most common, followed by the hepatic artery. Hepatic artery aneurysms are found in .02–0.4% of the population and are rarely symptomatic; however, hepatic artery aneurysms have the highest rate of spontaneous rupture,^6^ and rupture can be catastrophic. Most of these aneurysms are found incidentally on CT; they are commonly seen in male patients in their sixties who have atherosclerosis.^2^

Other risk factors include connective tissue disorders, fibromuscular dysplasia, multiparity, transplant of an abdominal organ, and portal hypertension. Pseudoaneurysms have also been reported in splanchnic arteries, but they are usually secondary to trauma, infection, inflammatory changes, or chronic pancreatitis. Symptomatic hepatic artery aneurysms that are > 2 cm, expand by more than 0.5 cm in a year, or those that are found in patients with a history of vasculopathy should be considered for repair.^7^ Techniques for repair range from open surgical intervention to endovascular techniques with IR.^1^,^8^ While open surgical repair had been considered the gold standard, recent advances in endovascular techniques and technology in conjunction with reported faster recovery, lower cost, and shorter length of stay, the endovascular approach is now recommended by the Society of Vascular Surgery as the primary intervention. Our patient received successful endovascular coil embolization. Hepatic and other splanchnic artery aneurysms are not commonly included in the differential of abdominal pain, but it should be considered.^3^ Rupture of such aneurysms should be considered in all those with concomitant hemodynamic instability.

## CONCLUSION

This case highlights a rare cause of abdominal pain associated with hemodynamic instability. The differential is wide including intra-abdominal infection and sepsis, abdominal aortic aneurysm rupture, traumatic organ damage, and aortic dissection. It is imperative to keep the differential broad and to include vascular etiologies, even those not commonly seen. Although point-of-care ultrasound was used initially in the evaluation of this patient, no intra-abdominal fluid was noted (which emphasizes the importance of operator expertise). While early recognition of hemoperitoneum may have prompted quicker evaluation by CT angiography, it would not have precluded it; the localization of the bleeding site remained imperative. The importance of interventional radiology and embolization as a stabilizing measure is paramount, especially in facilities that may lack surgical subspecialties capable of treating patients with splanchnic artery aneurysms.
